# CliqueFluxNet: Unveiling EHR Insights with Stochastic Edge Fluxing and Maximal Clique Utilisation Using Graph Neural Networks

**DOI:** 10.1007/s41666-024-00169-2

**Published:** 2024-08-01

**Authors:** Soheila Molaei, Nima Ghanbari Bousejin, Ghadeer O. Ghosheh, Anshul Thakur, Vinod Kumar Chauhan, Tingting Zhu, David A. Clifton

**Affiliations:** 1 Department of Engineering Science, University of Oxford, Oxford, OX1 3AZ UK; 2 Independent, Tehran, Iran; 3 Oxford-Suzhou Centre for Advanced Research (OSCAR), Suzhou, 215123 China

**Keywords:** Electronic health records, Graph neural network, Representation learning, Semi-supervised learning

## Abstract

Electronic Health Records (EHRs) play a crucial role in shaping predictive are models, yet they encounter challenges such as significant data gaps and class imbalances. Traditional Graph Neural Network (GNN) approaches have limitations in fully leveraging neighbourhood data or demanding intensive computational requirements for regularisation. To address this challenge, we introduce CliqueFluxNet, a novel framework that innovatively constructs a patient similarity graph to maximise cliques, thereby highlighting strong inter-patient connections. At the heart of CliqueFluxNet lies its stochastic edge fluxing strategy — a dynamic process involving random edge addition and removal during training. This strategy aims to enhance the model’s generalisability and mitigate overfitting. Our empirical analysis, conducted on MIMIC-III and eICU datasets, focuses on the tasks of mortality and readmission prediction. It demonstrates significant progress in representation learning, particularly in scenarios with limited data availability. Qualitative assessments further underscore CliqueFluxNet’s effectiveness in extracting meaningful EHR representations, solidifying its potential for advancing GNN applications in healthcare analytics.

## Introduction

Electronic Health Records (EHRs) are digital records of patient information collected and stored during medical encounters, such as demographics, diagnoses, and medications [1–3]. EHRs can provide longitudinal patient records that capture disease progression and treatment outcomes over time [4]. EHRs have been widely used for various applications in clinical research [5–7], large-scale observational studies [8], and clinical decision support systems [9–12]. However, EHRs also pose significant challenges for data modelling and analysis. One challenge is the high rate of missing and irregularly sampled data, which may result from different data collection practices and protocols in healthcare settings [13, 14]. For example, in the Medical Information Mart for Intensive Care (MIMIC-III) dataset, a widely used open-access EHR dataset, more than 80% of the data are missing [15, 16]. Another challenge is the class imbalance and under-representation of certain patient groups based on their diagnoses, which may affect the model performance and generalisability for these groups [17]. Therefore, there is a need for learning EHR representations that can handle missing and irregular data, and account for class imbalance and under-representation. Such representations could enable better use of EHR data for clinical research and decision support systems.

EHR data have a complex and rich hierarchical structure that reflects multiple levels of information and relationships among them. For example, a patient may have multiple visits over time, each visit may have multiple diagnoses, and each diagnosis may be associated with multiple medications or procedures. This hierarchical structure can provide valuable information for data analysis and prediction tasks, as it can capture the temporal and causal dependencies among the data elements [18]. However, most of the current machine learning models represent data in a tabular format, which flattens the hierarchical structure and disregards the inherent semantics of the data [19]. As a result, they might not be suitable for modelling nested or sequential data, such as visits, diagnoses, medications, and procedures [19].

To overcome this limitation, some recent studies have exploited non-tabular data formats, such as graphs or sequences, to model the hierarchical structure of EHRs using deep learning techniques, such as graph neural networks (GNNs) or recurrent neural networks (RNNs) [20, 21]. These studies claim to outperform tabular models for some tasks or datasets, by learning better representations of the EHR data while preserving some relational hierarchical properties [20, 22, 23]. However, these studies also have some limitations or challenges, such as computational complexity and generalisability [24]. For example, some studies rely on predefined ontologies which are not always available or applicable to different EHR data sources [25], while others use attention-based transformers to learn weights of the connections [21, 26], which could be computationally expensive [21] and not generalisable for data with high rates of missing data [26]. Therefore, there is still room for improvement in learning EHR representations that can capture the hierarchical structure of EHRs while addressing these challenges.

In this study, we introduce CliqueFluxNet, a novel graph-based model for EHRs designed to robustly and generatively capture patient data patterns. CliqueFluxNet innovatively constructs graphs from patient encounters, e.g., a single admission or visit to the ICU, emphasising clinical similarities without relying on fixed vocabularies or ontologies. A core feature of our model is the strategic use of *maximal cliques*, which enables the discovery of intricate relationships within the graph and enhances the network’s ability to identify nuanced patient similarities. This approach is inspired by studies demonstrating that clique-based models can efficiently learn when we have smaller training sets [27]. Furthermore, CliqueFluxNet employs an *adaptive edge-weighting scheme* based on these clique connections, combined with a sampling and aggregation mechanism, to effectively learn embeddings from neighbouring patient nodes. To enhance generalisability and prevent overfitting, especially in sparse data scenarios, our model incorporates *Edge Flux*, which is a randomisation strategy during training, adjusting edges to reflect potentially unobserved similarities. This comprehensive methodology, tested extensively on large-scale EHR datasets, demonstrates the superiority of CliqueFluxNet in predicting critical patient outcomes such as mortality and readmission. The clinical relevance of the learned embeddings and weights further underscores the practical implications of CliqueFluxNet in healthcare analytics, making it a valuable tool for accurate and reliable patient care decisions.

The primary contributions of our work are as follows:

- We introduce CliqueFluxNet, an inductive model that merges graph-based topological learning in EHRs through clique-based weighted computation. The dual approach of sampling and aggregating patient features from neighbouring nodes showcases a unique fusion of machine learning and healthcare informatics.
- The model includes a randomisation strategy during training, addressing potential unobserved similarities and preventing overfitting; enhancing its application in diverse healthcare analytics scenarios.
- Through rigorous testing across various scenarios, including mortality and readmission predictions on extensive EHR datasets, CliqueFluxNet demonstrates superior performance over existing state-of-the-art graph-based models. This is particularly notable in scenarios with limited training data, a common challenge in healthcare informatics. Our results highlight CliqueFluxNet’s robustness and versatility, setting new benchmarks in EHR data analysis.

We begin by reviewing related work in Section 2, followed by a detailed exposition of our methodology in Section 3. Section 4 presents our experimental results, showcasing the efficacy of CliqueFluxNet. Finally, Section 5 concludes with a discussion on the implications of our findings and potential avenues for future research.

## Related Works

This section provides a summary of related works in representation learning and GNNs for EHR applications.

### GNNs

In deep learning research, a key focus is extending neural networks to process graph data [28–30]. Two notable architectures within GNNs are the Graph Convolutional Network (GCN) [28] and Graph Attention Network (GAT) [31, 32]. While GCN generalises Convolutional Neural Networks (CNNs) to handle graph-structured inputs, GAT exploits attention framework to learn local features by assigning varied importance to nodes and attending to their neighbourhood features. Contemporarily, GraphSAGE introduced [29] focuses on inductive learning by sampling and aggregating information from the local neighbourhood of each node. It samples a fixed-size neighbourhood around each node, aggregates information from the sampled nodes, and then learns embeddings for the target node. On a different front, Deep Graph Infomax (DGI) [33] represents an unsupervised approach for learning graph representations through local–global information maximisation.

### Learning EHR Representations

Learning EHR representations has sparked broad interest within the research community, with numerous studies exploring various methods of embedding medical concepts [22, 34–37]. For instance, [38, 39] employed transformer-based models [40], integrating BERT [41] into their proposed models for medical records [38–40]. Conversely, a handful of works have delved into learning graphical representations of EHRs. For example, Multilevel Medical Embedding (MiME) [25] derived visit representations from the visit structure, surpassing a range of bag-of-features methods. Despite MiME’s promising results, the proposed approach is challenging to generalise, as it relies on a predefined external medical ontology to learn relationships across medical codes.

To overcome the limitation observed in MiME, Choi et al. combined the GCN [26] with a transformer to develop a graph-based representation model for EHRs. To address the challenge of transformers in effectively learning attention parameters, the authors integrated a predefined conditional probability matrix, derived from encounter records, to guide the attention derivation and regularisation process. The authors computed this matrix based on the co-occurrence relationship among medical concepts (diagnosis, treatments, and labs), which is later used to apply weights to the edges. Although this method surpassed baselines, relying on a predefined conditional probability in scenarios with high rates of missing data renders the strict definition of such probabilities and hierarchies non-generalisable. To address this limitation, Variational Graph Neural Network (VGNN) [21] was introduced by adding variational regularisation in its encoder-decoder graph network, enabling more generalised structural learning without predefined rules. While VGNN demonstrates enhanced performance across various prediction tasks, its approach is hampered by higher computational complexity.

## Preliminaries

We briefly introduce the preliminaries of Graph Networks in this section.

### GNNs

GNNs are advanced neural architectures specifically designed for processing data structured in graph form [42]. These networks are characterised by their unique ability to capture the complex relationships inherent in graph data. Central to a GNN is its representation of graph data as $({\textbf {X}}, {\textbf {A}})$: ${\textbf {X}} \in \mathbb {R}^{ N \times F }$ is the node feature matrix for *N* nodes, each with *F* features, and ${\textbf {A}} \in \mathbb {R}^{ N \times N }$ represents the adjacency matrix, encapsulating the inter-node connections. In a GNN, each layer is designed to refine and elevate the node features. Starting from the initial feature representation ${\textbf {X}}={\textbf {H}}^{(0)}$, a GNN layer processes the current feature matrix ${\textbf {H}}^{(l-1)} \in \mathbb {R}^{ N \times F }$ along with ${\textbf {A}}$ to produce an evolved feature representation ${\textbf {H}}^{(l)} \in \mathbb {R^{ N \times F }}$, as described by:

1 $$\begin{aligned} {\textbf {H}}^{(l)}=f({\textbf {H}}^{(l-1)},\; {\textbf {A}}). \end{aligned}$$

Among the diverse GNN architectures, the GCN stands out as a prominent transductive GNN encoder, $\mathcal {E}$. The GCN updates node features via a sophisticated layer-wise rule:

2 $$\begin{aligned} \begin{array}{cc} \mathcal {E}({\textbf {X}}, {\textbf {A}}) = \sigma \left( \hat{{\textbf {D}}}^{-\frac{1}{2}}\hat{{\textbf {A}}}\hat{{\textbf {D}}}^{-\frac{1}{2}}  {\textbf {X}}\varvec{\Theta }\right) \end{array} \end{aligned}$$

Here, $\sigma$ denotes the ReLU activation function. The matrix $\hat{{\textbf {A}}}= {\textbf {A}} + {\textbf {I}}_N$ is an enhanced adjacency matrix, augmented with self-loops via an $N \times N$ identity matrix ${\textbf {I}}_N$. The degree matrix $\hat{{\textbf {D}}}_{ii}= \sum _{j} \hat{{\textbf {A}}}_{ij}$ and the transformation matrix $\varvec{\Theta } \in \mathbb {R^{ F \times F^\prime }}$ work in tandem to dynamically update each node’s features through the learning process, typically optimised via a back-propagation algorithm minimising a chosen loss function (e.g., cross-entropy loss) [28].

### Cliques in Graphs

To better understand the components of the proposed work, we provide an overview of important terminology and definitions in graph and GNN theory.

#### Definition 1

Provided a graph $G = ({\textbf {V}}, {\textbf {E}})$ with ${\textbf {V}}$ nodes and ${\textbf {E}}$ edges, a clique is a subset $C \in {\textbf {V}}$ in which every node is adjacent to all other nodes in the set, $(v, v^{\prime }) \in {\textbf {E}}, \forall v, v^{\prime } \in C$ [43].

#### Definition 2

In a graph, a maximal clique cannot be expanded by incorporating one more neighbouring node without affecting the clique’s connectedness [44].

#### Listing Maximal Cliques

The Bron-Kerbosch method [45] has been used in this work to list maximal cliques. This method operates by managing three distinct sets of nodes: *Q*, *P*, and *R*. Here, nodes in set *Q* are candidates for removal from the clique, while set *P* contains potential candidates to augment clique *R*. Set *R* represents the evolving clique under construction. In each recursive call, the method selects a node *v* in *P* to join the clique *R*, and when the recursive call returns *v* is pushed to *Q*. *R* is returned as a maximal clique when *P* and *Q* are both empty.

## Methodology

**Fig. 1:**
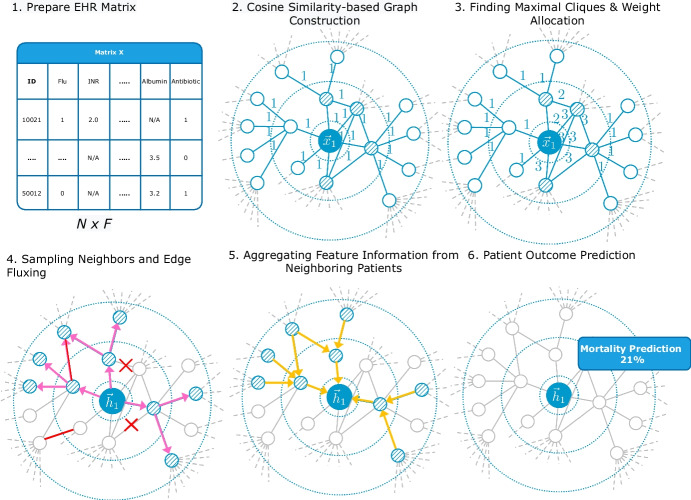
Overview of the proposed model, CliqueFluxNet. Starting with a prepared EHR matrix, our model starts a graph construction based on the cosine similarity between encounters. Finding maximal cliques and weight allocation are conducted using Bron-Kerbosch algorithm. Steps 4 (pink arrows) and 5 (yellow arrows) represent the node sampling and feature aggregation, where random edge deletion and addition (in red) are conducted throughout the training. Directed edges introduced in steps 4 and 5 of the process serve solely to illustrate sampling and aggregation procedures in graph representation learning. Lastly, the patient outcome predictions are based on the learnt aggregated representations. Edge deletion and addition are shown in red. Best viewed in colour

This section describes the proposed CliqueFluxNet framework for EHR representation learning in detail. Figure 1 presents a visual depiction of the framework’s architecture, offering a clear overview of its components and interactions.

### Overview of CliqueFluxNet

Assuming we are provided with a set of encounters that are represented by a feature matrix, ${\textbf {X}} \in \mathbb {R}^{N \times F}$ where *N* is the number of patients and *F* is the feature dimensions, we outline CliqueFluxNet as follows:

1. CliqueFluxNet begins by constructing a graph from encounters, represented by ${\textbf {X}}$, using cosine similarities between encounters. This graph construction is pivotal for understanding the complex relationships in the EHR data.
2. The Bron-Kerbosch algorithm is applied to list all maximal cliques within this graph, a crucial step that allows our model to focus on strongly connected nodes. This process updates the adjacency matrix ${\textbf {A}}$, enabling an intricate representation of patient encounters based on their clique membership.
3. The proposed method frames patient outcome prediction as a node classification problem. We derive high-level representations for each encounter using an encoder $\mathcal {E}({\textbf {X}}, {\textbf {A}})$, leveraging mean-pooling layers to perform this transformation.
4. During training, we introduce a graph randomisation strategy, Edge Flux, adding or deleting edges randomly to accommodate potential unobserved similarities. This step ensures the robustness and adaptability of our model to various EHR data scenarios.
5. Finally, the training objective is defined using a binary cross-entropy loss function, optimising the network’s ability to predict patient outcomes accurately.

### Notations

We refer to the constructed graph as *G*, which is shown as $({\textbf {X}}, {\textbf {A}})$, where ${\textbf {X}} \in \mathbb {R}^{N \times F}$ indicates the matrix of node features with *N* nodes and *F* features per node, and ${\textbf {A}} \in \mathbb {R}^{N \times N}$ is the adjacency matrix, which corresponds to the similarities between patient nodes. Nodes must be assigned to one of the *y* target classes. Moreover, we assume directed and unweighted graphs, i.e., ${\textbf {A}}{ij}=0$ if there is no edge between nodes *j* and *i* and ${\textbf {A}}{ij} = 1$ otherwise.

### CliqueFluxNet

A set of *N* encounters, represented as $\textbf{X} = \lbrace \vec {x}_1, \vec {x}_2, \ldots , \vec {x}_N \rbrace$, is provided as input, where $\vec {x}_i \in \mathbb {R}^F$ represents the features of the *i*-th encounter. The relational information between these encounters, $\textbf{A} \in \mathbb {R}^{N \times N}$, is not provided as input, as EHR datasets often do not contain this information [25]. The proposed framework first constructs the graph using the set of encounter features based on cosine similarity [46] among encounters. We formally define cosine similarity, $k(\vec {x}_i, \vec {x}_j)$, between two encounters represented by their feature vectors $\vec {x}_i$ and $\vec {x}_j$, as follows:

3 $$\begin{aligned} k(\vec {x}_i, \vec {x}_j) = \frac{\vec {x}_i \cdot \vec {x}_j^{\top }}{\Vert \vec {x}_i \Vert \cdot \Vert \vec {x}_j \Vert } \end{aligned}$$

where the symbol $\cdot$ denotes the dot product of the feature vectors, with $\top$ representing the transpose operation. Additionally, $|\vec {x}_i|$ and $|\vec {x}_j|$ represent the Euclidean norms of vectors $\vec {x}_i$ and $\vec {x}_j$, respectively. Two encounters are connected by an edge if the cosine similarity between them is greater than 0.85.

Subsequently, the proposed framework lists maximal cliques from the constructed graph in different hops to learn useful representations, as used by [27, 47]. The Bron-Kerbosch method [45] is used to obtain all maximal cliques in the graph. Then, the adjacency matrix is updated such that ${\textbf {A}}_{ij} = c$ indicates encounters *j* and *i* belong to a $(c + 1)$-clique. This allows the framework to focus on nodes with strong connections while aggregating information from the neighbouring nodes.

Having constructed the patient encounter graph and allocated weights to different edges in a neighbourhood, we frame the patient outcome prediction task as a node classification problem. We learn an encoder, $\mathcal {E}({\textbf {X}}, {\textbf {A}})= \left\{ \vec {h_1}, \vec {h_2}, \ldots , \vec {h_N} \right\}$, such that $\vec {h_i} \in \mathbb {R}^{F^{\prime }}$ represents high-level representations for the *i*-th encounter and $\mathcal {E}: \mathbb {R}^{N \times N} \times \mathbb {R}^{N \times F} \rightarrow \mathbb {R}^{N \times F^{\prime }}$. Finally, these representations corresponding to patient encounters can be used for outcome prediction problems.

This work employs an encoder that is built on the mean-pooling (MP) [29], defined as follows:

4 $$\begin{aligned} MP({\textbf {X}}, {\textbf {A}})=\sigma \left( \hat{{\textbf {D}}}^{-1} \hat{{\textbf {A}}} {\textbf {X}} \varvec{\Theta }\right) , \end{aligned}$$

where $\sigma$ denotes the ReLU activation, $\hat{{\textbf {A}}}$ is enhanced adjacency matrix, $\hat{{\textbf {D}}}$ is degree matrix, and $\varvec{\Theta }$ represents the trainable transformation matrix as described in Section 3.1.

The encoder is a two-layered mean-pooling as described below:

5 $$\begin{aligned} \mathcal {E}({\textbf {X}}, {\textbf {A}})=MP_2(MP_1({\textbf {X}}, {\textbf {A}}), {\textbf {A}}). \end{aligned}$$

Each MP layer generates 16-dimensional features ($F^\prime =16$). During training, we employ an *Edge Flux* strategy where the input graph is deformed by stochastically adding or deleting edges in order to account for potential similarities that might not be observed in the EHR. As per Edge Flux, for each pair of nodes $(i, j)$, the updated adjacency matrix $\textbf{A}'$ is given by:

6 $$\begin{aligned} \textbf{A}'_{ij} = {\left\{ \begin{array}{ll} 1 &  \text {with probability } p_{\text {add}} \text { if } \textbf{A}_{ij} = 0, \\ 0 &  \text {with probability } p_{\text {delete}} \text { if } \textbf{A}_{ij} = 1, \\ \textbf{A}_{ij} &  \text {otherwise}. \end{array}\right. } \end{aligned}$$

Here $p_{\text {add}}$ is the probability of adding an edge where there is none and $p_{\text {delete}}$ is the probability of deleting an existing edge.

Edge Flux can be applied once per training epoch to introduce randomness into the graph structure. For the training objective, we use a standard binary cross-entropy (BCE) loss between the target and predicted labels:

7 $$\begin{aligned} \mathcal {L}=-\frac{1}{N} \sum _{i=1}^{N} \left( \hat{y_i} \log \left( y_i\right) + \left( 1-\hat{y_i}\right) \log \left( 1 -y_i \right) \right) , \end{aligned}$$

where $\hat{y_i}$ is the network’s predicted label and $y_i$ is the ground-truth label.

### Datasets and Preprocessing

#### Datasets

The proposed framework is evaluated on two publicly available large EHR datasets: MIMIC-III and eICU.

##### MIMIC-III

MIMIC-III [15] is a freely accessible de-identified database containing data for adult patients (aged 16 or older) hospitalised in critical care units. This dataset was collected between 2001 and 2012 at Beth Israel Deaconess Medical Centre (BIDMC) in the United States. This dataset contains information regarding demographics, patient outcomes, and vital signs, as well as medical procedures and medications. More details can be found in [15].

##### eICU

The Philips eICU Collaborative Research Dataset [48] is a multi-centre dataset that includes 200,859 patient encounters for 139,367 unique patients hospitalised between 2014 and 2015 to one of 335 units at 208 hospitals across the United States. eICU has been used for many healthcare research applications, particularly for studies investigating the development and validation of models across multiple centres [49, 50].

#### Preprocessing

We adopt the preprocessing method proposed by [26] to derive EHR representations from both the MIMIC and eICU datasets. To preprocess the MIMIC and eICU datasets, we exclude encounters lasting less than 24 h and remove duplicate treatment codes (e.g., medications administered repeatedly). Additionally, we omit lab results due to their potential fluctuation over time in an ICU setting (e.g., blood pH level). As a result, we retained 50, 391 encounters from MIMIC and 41, 026 encounters from eICU. Utilising CliqueFluxNet, we obtain representations for each encounter, which we then apply to predict patient outcomes. Throughout this study, we overlook the time-series aspect of EHRs and concentrate on individual encounters. Table 1 presents the statistical breakdown of the datasets employed for training and evaluating CliqueFluxNet.

In our data preprocessing pipeline, we handle missing values using a combination of filtering and imputation techniques. We process the EHRs as follows:

- Filtering: We filter out encounters with a duration exceeding a specified threshold (e.g., 24 h) to focus on relevant data points.
- Imputation: In scenarios where missing values are encountered, we exploited mean imputation for numerical features and mode imputation for categorical features.

**Table 1:** Statistical characteristics of the preprocessed datasets used to train and evaluate the model for both the mortality and readmission tasks

Dataset	MIMIC	eICU
Avg. # of diagnosis per visit	11.5	6.5
Avg. # of treatment per visit	4.5	5.0
# of Positives (Readmission)	N/A	7,051
# of Positives (Mortality)	5,377	2,983
# of Encounters	50,391	41,026

### Baselines and Experimental Settings

#### Baselines

To evaluate our model in downstream prediction tasks, we compare its performance concerning multiple baselines, including state-of-the-art graph learning models on EHRs:

- **Random Forest (RF):** an ensemble of decision trees where each tree is built based on data samples from training sets with replacement [51].
- **Multi-Layer Perceptron (MLP):** a model that uses a stack of linear layers with ReLU activation after each layer, with the exception of the final one, where softmax activation is used to make the predictions [52].
- **Graph Convolutional Transformer (GCT):** a graph-based model that uses a Transformer to learn the representation of EHRs. The model leverages conditional probabilities resulting from the correlation between medical concepts to guide regularisation of the attention [26].
- **VGNN:** an encoder-decoder graph network with variational regularisation to learn the similarities among patient nodes [21].

#### Prediction Tasks

We train and evaluate CliqueFluxNet and baseline models in predicting two main tasks.

- **Mortality prediction:** We train models using patient encounter data to predict a binary outcome indicating mortality. This task is evaluated on both eICU and MIMIC datasets.
- **Readmission prediction:** Using the patient encounter data, we evaluate the baseline models in predicting whether each patient will be readmitted to the ICU again during the same hospital stay. This task is only evaluated on the eICU dataset.

#### Training Setup

We train CliqueFluxNet using Adam optimiser with a fixed learning rate of 0.001, batch size of 128 and cross-entropy loss for 250 epochs. The area under the Precision-Recall curve (AUPRC) [53] is used as a performance metric. The choice of AUPRC as a metric stems from the imbalanced nature of the patient outcomes in EHR datasets. We evaluate the proposed framework on four different train, validation and test splits: $\{(70\%:15\%:15\%), (40\%:30\%:30\%), (30\%:35\%:35\%), \text {and}\; (20\%:40\%:40\%)\}$. These splits are used to evaluate the proposed framework on both data-rich and data-scarce scenarios. Note that patients used for training are not involved in validation or testing across all experimental settings. We employ the best AUPRC over the validation datasets to select the model configuration final evaluation. The performance evaluation is repeated 5 times by randomly sampling training, validation, and test sets.

**Fig. 2:**
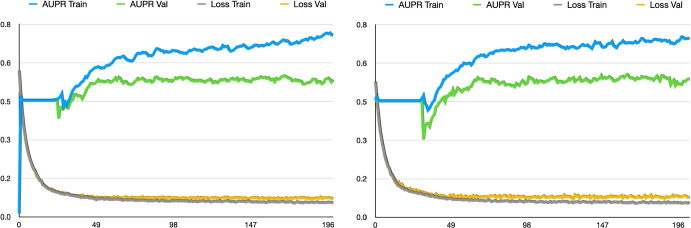
AUPRC and loss during training of CliqueFluxNet on the MIMIC (**left**) and eICU (**right**) datasets

**Table 2:** Readmission and mortality prediction performance on eICU and MIMIC in terms of AUPRC. Bold entries represent the best performing method in each setting

Settings	Models	MIMIC Mortality	eICU Mortality	eICU Readmission
70%:15%:15%	RF [51]	0.5524$\pm {0.011}$	0.4992$\pm {0.005}$	0.4528$\pm {0.012}$
	MLP [52]	0.5231$\pm {0.026}$	0.4321$\pm {0.035}$	0.4419$\pm {0.015}$
	GCT [26]	0.5812$\pm {0.025}$	0.5733$\pm {0.026}$	0.4827$\pm {0.015}$
	VGNN [21]	0.5927$\pm {0.027}$	**0.5964±0.027**	0.5795$\pm {0.017}$
	CliqueFluxNet	**0.5972±0.025**	0.5939$\pm {0.026}$	**0.5802±0.016**
40%:30%:30%	RF [51]	0.5124$\pm {0.022}$	0.4737$\pm {0.021}$	0.4263$\pm {0.012}$
	MLP [52]	0.5102$\pm {0.027}$	0.4684$\pm {0.028}$	0.4271$\pm {0.015}$
	GCT [26]	0.5672$\pm {0.023}$	0.5136$\pm {0.028}$	0.4773$\pm {0.017}$
	VGNN [21]	0.5792$\pm {0.025}$	0.5515$\pm {0.026}$	0.4819$\pm {0.019}$
	CliqueFluxNet	**0.5952±0.025**	**0.5739±0.024**	**0.5243±0.015**
30%:35%:35%	RF [51]	0.5025$\pm {0.018}$	0.4528$\pm {0.019}$	0.3976$\pm {0.014}$
	MLP [52]	0.4812$\pm {0.033}$	0.4326$\pm {0.030}$	0.3873$\pm {0.026}$
	GCT [26]	0.5429$\pm {0.021}$	0.5275$\pm {0.024}$	0.4556$\pm {0.014}$
	VGNN [21]	0.5629$\pm {0.026}$	0.5386$\pm {0.029}$	0.4718$\pm {0.018}$
	CliqueFluxNet	**0.5994±0.025**	**0.5554±0.022**	**0.5129±0.017**
20%:40%:40%	RF [51]	0.5127$\pm {0.019}$	0.4562$\pm {0.020}$	0.4248$\pm {0.012}$
	MLP [52]	0.4364$\pm {0.028}$	0.4273$\pm {0.028}$	0.4018$\pm {0.031}$
	GCT [26]	0.5183$\pm {0.022}$	0.4883$\pm {0.021}$	0.4882$\pm {0.013}$
	VGNN [21]	0.5472$\pm {0.030}$	0.4884$\pm {0.024}$	0.4913$\pm {0.032}$
	CliqueFluxNet	**0.5723±0.023**	**0.5386±0.022**	**0.5202±0.014**

## Results

### Predictive Performance

Figure 2 portrays the training dynamics of the proposed CliqueFluxNet framework, trained for mortality prediction on both the MIMIC-III and eICU datasets using a 70%:15%:15% split. Examination of this figure underscores the framework’s ability to effectively train for the designated tasks, as indicated by the diminishing training and validation losses with training progression.

Table 2 showcases the performance comparison between CliqueFluxNet and the baseline methods. In a standard setting with a 70%:15%:15% split, our framework consistently outshone nearly all baseline approaches, yielding an impressive average AUPRC of 0.5972 and 0.5939 for mortality prediction on the MIMIC and eICU datasets, respectively. Notably, our framework demonstrated comparable performance to the top-performing VGNN baseline in predicting mortality on the eICU dataset. CliqueFluxNet also surpassed all baselines, achieving an effective AUPRC score of 0.5802 for predicting readmissions.

**Fig. 3:**
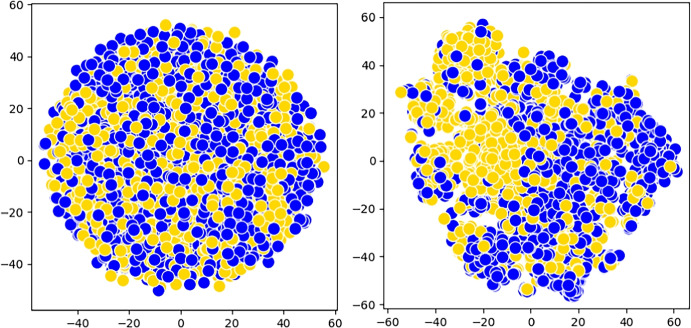
t-SNE embeddings of the patients in the eICU dataset based on raw (**left**) and learnt CliqueFluxNet features (**right**). Yellow points represent readmitted individuals, while blue points represent not-readmitted individuals. Best viewed in colour

To evaluate the performance of the proposed framework in data-scarce scenarios, we train baselines and the proposed framework using data splits with fewer training examples. When decreasing the training examples to $40\%$, $30\%$, and $20\%$ of the available dataset, we observed a significant decline in the performance of baseline methods. For instance, reducing the training examples from $70\%$ to $20\%$ results in a relative drop of $7.67\%$ in the average performance of VGNN, the best-performing baseline, for MIMIC-III mortality prediction. However, CliqueFluxNet maintained a consistent and robust performance even when the training data was reduced. In all three tasks, the performance of CliqueFluxNet experienced a notably smaller relative drop compared to the baseline methods. For instance, in the MIMIC-III mortality task, CliqueFluxNet achieved a comparable AUPRC of 0.5723 even with only $20\%$ of the training examples. Thus, the proposed framework can be considered data-efficient and has produced effective EHR representations, resulting in strong performance across all experimental settings.

**Fig. 4:**
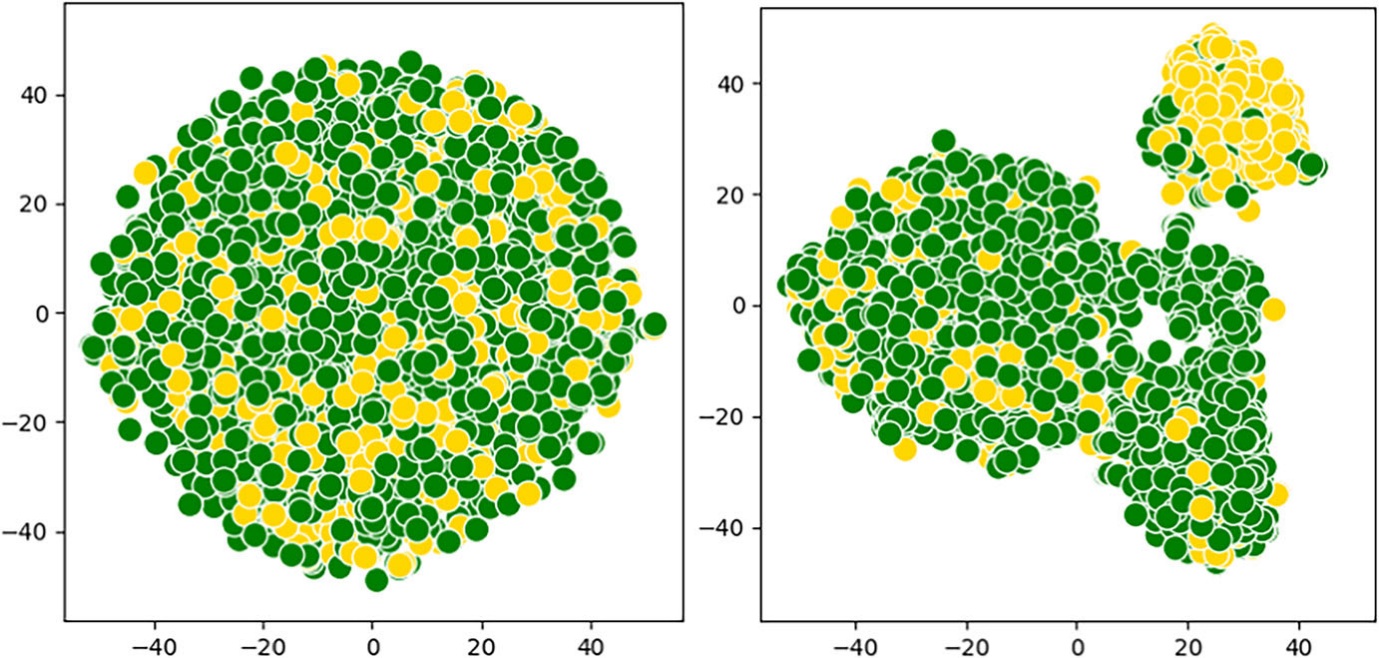
t-SNE embeddings of the patients in the MIMIC dataset based on raw (**left**) and learnt CliqueFluxNet features (**right**). Yellow points represent patients with a positive mortality label, while green points represent patients with a negative mortality label. Best viewed in colour

subsectionQualitative Analysis To underline the efficacy of CliqueFluxNet and the quality of representations, we plot a set of t-distributed stochastic neighbour embedding (t-SNE) plots [54] of the produced representations via CliqueFluxNet for mortality prediction task (Fig. 4) using the MIMIC dataset and readmission prediction task (Fig. 3) using the eICU dataset, respectively. The different colours denote different patient classes. Note that these classes correspond to the labels of the datasets, verifying the model’s discriminative power across the binary patient outcome prediction tasks. We perform further analyses to demonstrate t-SNE embeddings of the patients based on the learnt features from GCT and VGNN models. These qualitative outcomes can be seen in the Appendix. The qualitative results demonstrate the model’s ability to learn representations where patients with similar outcomes are close to each other (Fig. 4).

**Fig. 5:**
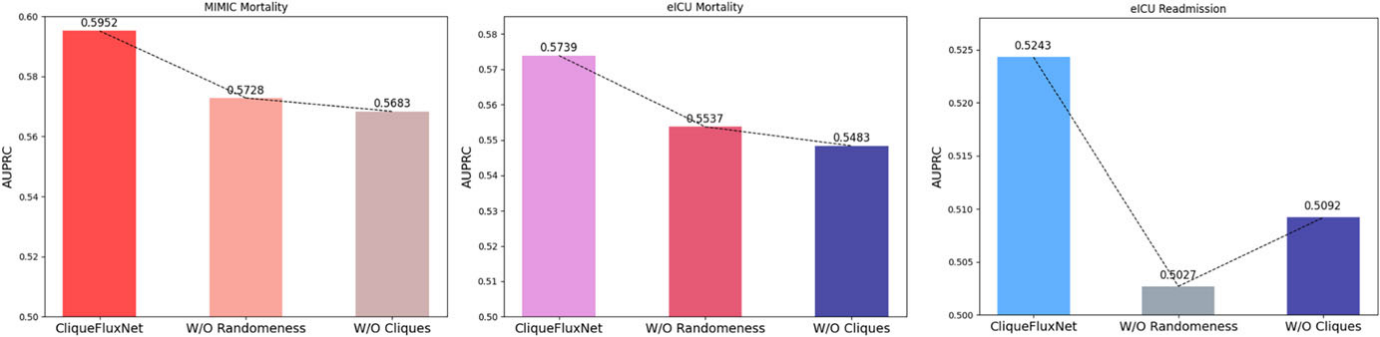
Performance of the CliqueFluxNet in the absence of randomness and clique components in terms of AUPRC

## Ablation Study

An ablation study was conducted to assess the performance of CliqueFluxNet in the absence of Edge Flux, i.e., random edge addition and deletion (CliqueFluxNet W/O Edge Flux), as well as without maximal cliques-based edge weighting (CliqueFluxNet W/O Clique). The results, measured in terms of AUPRC with a 40%:30%:30% data split, are presented in Fig. 5. The analysis of this figure highlights that the removal of Edge Flux as well as clique-based weighting results in a noticeable drop in performance across all three tasks. This confirms the importance of Edge Flux and clique-based weighting in regularising the training, learning generalised EHR representations, and capturing intricate inter-patient relationships.

Upon analysing Fig. 5 alongside Table 2, it is clear that CliqueFluxNet W/O Edge Flux outperforms all baselines except VGNN across all three tasks. Similar behaviour is observed for CliqueFluxNet W/O Clique. This further affirms the importance of both Edge Flux and clique-based weighting in achieving better performance than VGNN and other baselines.

## Discussion and Conclusion

In the study, a novel graph-based topological structure rooted in patient similarity was introduced. This structure facilitated the extraction of all maximal cliques within the graph, thereby enabling the acquisition of high-level patient representations. Through sampling and aggregating feature information from patients in their local neighbourhoods, the model was designed to flexibly capture the dynamic and heterogeneous nature of EHRs.

The proposed model, CliqueFluxNet, underwent rigorous training and validation using two extensive EHRs datasets, covering three distinct tasks, which included two mortality prediction challenges and a readmission prediction task. Comparative evaluations against robust baselines underscored the superior performance of CliqueFluxNet, particularly evident in the AUPRC. The resilience of CliqueFluxNet stems from its ability to sample and aggregate neighbouring patients, facilitating effective similarity detection even with limited training data. A crucial aspect of the framework is the edge-weighting component, which assigns higher weights to stronger cliques. This enhances model robustness, especially in scenarios characterised by class imbalance and limited training data. The flexibility, independence from predefined relationships, and data-driven approach equip the proposed framework for handling diverse healthcare data scenarios.

Nonetheless, this study is not without its limitations. Our focus was primarily on three patient outcomes in the MIMIC and eICU datasets, thus overlooking various patient care scenarios where EHR representations can play an important role. Future endeavours will entail broadening our analysis to incorporate these aspects while also tackling the time-series nature inherent in EHRs.

In conclusion, CliqueFluxNet’s mastery of EHR representations represents a notable advancement beyond existing state-of-the-art graph models. Its effectiveness and resilience not only highlight its potential but also invite further exploration into graph-based representations across diverse healthcare applications. By pushing the boundaries of innovation in EHR analysis, CliqueFluxNet establishes a promising precedent for advancing patient care, clinical decision-making, and medical research. Its robust performance sets the stage for future efforts aimed at uncovering deeper insights and improving the efficacy of patient care models.

In conclusion, our approach, CliqueFluxNet, exceeds state-of-the-art graph models in learning EHR representations. The effectiveness and resilience of our model may pave the way for further research in graph-based representations across diverse healthcare applications.

## Figures and Tables

**Fig. 6:**
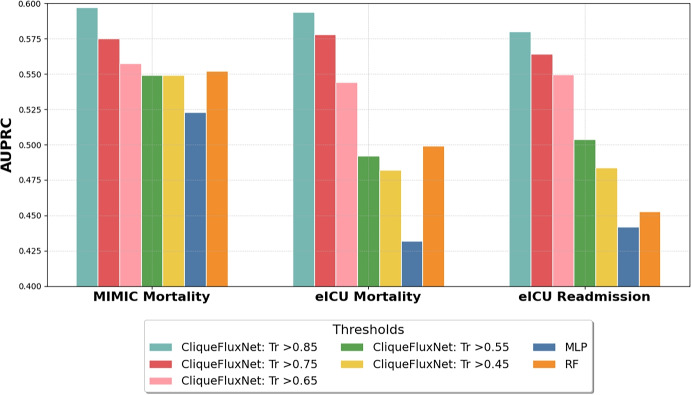
Performance comparison of predictive models at various thresholds for MIMIC Mortality, eICU Mortality, and eICU Readmission, measured by the AUPRC. Each bar represents the AUPRC score for a specific threshold

## Data Availability

No datasets were generated or analysed during the current study.

## Appendix A: Theoretical Justification of Random Edge Flux in GNNs

Assume a GNN model *f*, parameterised by $\theta$, that maps graph structures *G* to outputs. The generalisation error, which measures the model’s performance on unseen data, is defined as follows:

A1 $$\begin{aligned} \text {Generalisation Error} = \mathbb {E}_{G \sim D}[\mathcal {L}(f(G; \theta ), Y)] - \mathcal {L}(f(G_{\text {train}}; \theta ), Y_{\text {train}}) \end{aligned}$$

where $\mathcal {L}$ denotes the loss function, *D* is the distribution of graphs, and *Y* are the target outputs. Random edge deletion and addition are modelled as independent Bernoulli processes:

A2 $$\begin{aligned} P(\text {edge deletion})&= p_{\text {delete}},&P(\text {edge preservation})&= 1 - p_{\text {delete}}, \end{aligned}$$

A3 $$\begin{aligned} P(\text {edge addition})&= p_{\text {add}},&P(\text {no edge addition})&= 1 - p_{\text {add}}. \end{aligned}$$

These stochastic processes introduce variability in the graph structure, aiming to prevent overfitting by not allowing the model to rely too heavily on specific edges. To balance model complexity and performance, we introduce a regularised loss function:

A4 $$\begin{aligned} \mathcal {L}_{\text {reg}}(f(G; \theta ), Y) = \mathcal {L}(f(G; \theta ), Y) + \lambda R(\theta ), \end{aligned}$$

where $R(\theta )$ is a regularisation term that penalises complexity, and $\lambda$ is a coefficient determining the strength of regularisation. The expected smoothing effect of Random Edge Flux is quantified as follows:

A5 $$\begin{aligned} \mathbb {E}[\Vert f(G; \theta ) - f(G'; \theta ) \Vert _2] \le \epsilon , \end{aligned}$$

indicating that modifications to the graph structure should not drastically alter the model’s output, thereby ensuring stability. Here, $\epsilon$ represents a small, acceptable bound on the change in output due to graph perturbations.

The model’s stability to structural perturbations in the graph is further defined as follows:

A6 $$\begin{aligned} \text {If } G' \text { is a perturbation of } G, \text { then } |f(G; \theta ) - f(G'; \theta )| \le \delta , \end{aligned}$$

where $\delta$ denotes a small tolerance level for changes in the model output. Both $\epsilon$ and $\delta$ are positive, small quantities that ensure the model’s outputs are robust to minor changes in the graph structure.

## Appendix B: Mathematical Justification for Clique-Enhanced Adjacency Matrix

### 2.1 Introduction to Graph Properties and Clustering Coefficient

The clustering coefficient $C_i$ for a node $i$ quantifies how close its neighbours are to be a complete clique. In a standard adjacency matrix $A$, where $A_{ij} = 1$ signifies an edge between nodes $i$ and $j$, the clustering coefficient for node $i$ is defined as follows:

B7 $$\begin{aligned} C_i = \frac{2T(i)}{deg(i)(deg(i)-1)} \end{aligned}$$

Here, $T(i)$ represents the number of triangles involving node $i$, and $deg(i)$ is the degree of $i$, indicating the number of direct connections to $i$. For the modified context, where a clique-enhanced adjacency matrix $A_{\text {clique}}$ is considered, we define the enhanced clustering coefficient $C_i'$. This matrix emphasises maximal cliques and increases $T(i)$ for nodes within cliques. Therefore, the enhanced clustering coefficient $C_i'$ is given by:

B8 $$\begin{aligned} C_i' = \frac{2T_{\text {clique}}(i)}{deg_{\text {clique}}(i)(deg_{\text {clique}}(i)-1)} \end{aligned}$$

where $T_{\text {clique}}(i)$ accounts for the number of triangles that form part of maximal cliques involving node $i$, and $deg_{\text {clique}}(i)$ is the degree of $i$ within the context of the clique-enhanced graph. A higher $C_i'$ suggests denser local connections due to the influence of cliques.

### 2.2 Impact on Feature Aggregation in GNNs

Feature aggregation in GNNs is crucial for learning node representations. The aggregation for a node $i$ at layer $l+1$, considering its neighbourhood $\mathcal {N}(i)$, is enhanced in $A_{\text {clique}}$:

B9 $$\begin{aligned} h_i^{l+1} = \text {ReLU}\left( W^l \sum _{j \in \mathcal {N}(i)} \frac{1}{|\mathcal {N}(i)|} h_j^l + B^l h_i^l \right) \end{aligned}$$

The clique-enhanced matrix enhances the neighbourhood $\mathcal {N}(i)$ by incorporating clique-based connections, potentially enriching the aggregated features $h_i^{l+1}$ with more relevant information.

## Appendix C: Computational Complexity

The overall computational complexity of the proposed framework is dependent on computational complexity required for graph construction, identifying cliques, node encoding operation, edge flux and neighbourhood sampling operations. The graph construction through pairwise cosine similarities imposes a complexity of $O(n^2 \times d)$, with $n$ and $d$ representing the count of encounters (nodes) and their dimensional features in $\textbf{X}$, respectively. The complexity to compute maximal cliques using optimised Bron–Kerbosch algorithm is $O(m+n)$, where *m* is the number of edges. Node encoding operations, conducted by $\mathcal {E}(\textbf{X}, \textbf{A})$, maintain a complexity of $O(n^2)$, approximately. Integration of the Edge Flux strategy, i.e., modifying $m$ edges has a complexity of $O(m)$. Similarly, the neighbourhood sampling to update node representations has a complexity of $O(n \times d \times S)$, where $S$ is the number of sampled neighbours. Hence, the overall complexity is $O(n^2 \times d)$.

## Appendix D: Analysis for Various Thresholds

In the comparative analysis presented for 75:15:15 setting, the bar chart illustrates the AUPRC performance across various thresholds for MIMIC Mortality, eICU Mortality, and eICU Readmission. It is evident that for thresholds under 0.55, the AUPRC remains relatively stable, suggesting a plateau in performance improvement (Fig. 6). The best performance is observed for a threshold of 0.85 across all datasets.

### 4.1 Limitations

CliqueFluxNet, like many graph-based models, relies on the quality and clarity of the connections between patient data points. In cases where the EHR data is particularly noisy, the model may not effectively distinguish relevant patterns, which could lead to performance degradation. Under these circumstances, CliqueFluxNet’s performance might revert to being comparable to more traditional models like MLP, which do not utilise the complex network-based interactions between features. We can simulate these noisy scenarios by using a lower threshold of 0.55 or 0.45 in CliqueFluxNet resulting in spurious edges as we would expect in noisy conditions. At these thresholds, the performance of CliqueFluxNet is comparable to MLP as illustrated in Fig. 6.
